# Mesoporous Silica Nanoparticles Loaded with Resveratrol Are Used for Targeted Breast Cancer Therapy

**DOI:** 10.1155/2022/8471331

**Published:** 2022-09-19

**Authors:** Yifan Gu, Zhewei Fei

**Affiliations:** ^1^Department of Breast Surgery, Xinhua Hospital, Shanghai Jiaotong University School of Medicine, Shanghai 200092, China; ^2^Department of General Surgery, Xinhua Hospital Chongming Branch, School of Medicine, Shanghai Jiaotong University, Shanghai 202150, China

## Abstract

**Objective:**

The characteristics of poor pharmacokinetics, stability, and low solubility seriously limited the clinical application of resveratrol (Res) in breast cancer. Thus, this study intends to develop a delivery system for Res which could be better used in breast cancer therapy.

**Methods:**

Resveratrol-modified mesoporous silica nanoparticles (MSN-Res) were chemically constructed. Their shape and encapsulation were detected by transmission electron microscope, Fourier transforms infrared spectrometer, and UV spectroscopy, respectively. MGF-7 tumor-bearing mice were established by subcutaneous injection, and the pathological changes were detected by hematoxylin-eosin staining. CCK-8 and Ki-67 immunohistochemical staining were used for proliferation evaluation *in vitro* and *in vivo*. Flow cytometry, TUNEL, wound healing, and transwell assay detected cell apoptosis, invasion, and migration.

**Results:**

MSN-Res was successfully prepared with high biosafety. MSN-Res inhibited MGF-7 cell proliferation, invasion, and migration and promoted apoptosis *in vitro*. Furthermore, MSN-Res showed better performance compared Res in breast cancer mouse models. In addition, we found that MSN-Res inhibited tumor growth via inhibiting the NF-*κ*B signaling pathway.

**Conclusion:**

MSN-Res inhibited breast cancer progression with better efficacy compared with Res treatment alone by inhibiting the NF-*κ*B signaling pathway, suggesting that MSN-Res is a more effective adjuvant treatment method for breast cancer. Thus, our findings may provide a new and safer means of using phytochemicals in combinatorial therapy of breast cancer.

## 1. Introduction

Breast cancer is one of the most common female malignancies globally, accounting for 1/4 of the total number of new cancers in women, and the mortality rate accounts for 15% of female cancers. It seriously threatens the lives and health of women and has a tremendous influence on economic, social, and family problems [1]. Surgery is a recognized radical treatment for breast cancer, but surgery alone cannot effectively improve the prognosis of breast cancer patients [2, 3]. The recurrence and metastasis of cancer cells are the main factors leading to poor prognosis. Radiotherapy and chemotherapy can effectively increase the local control rate and improve the long-term survival rate, which is an essential auxiliary method for the clinical treatment of breast cancer [4]. However, long-term radiotherapy and chemotherapy have serious side effects and significantly increase the resistance of tumor tissues to radiotherapy and chemotherapy [5]. Systemic chemotherapies which are lack of specific targeting lead to significant healthy tissue damage [6]. Chemotherapy employs common therapeutic drugs with a 20% response rate achievement, but it also involves increased chemical resistance, systemic toxicity, and significant collateral damage leading to myelosuppression, immunosuppression, cardiotoxicity, neuropathy, and myalgia [7]. Therefore, more effective adjuvant treatment methods need to be explored.

Resveratrol (Res) is a natural polyphenol active ingredient extracted from plants, with antitumor and anti-aging effects. Studies have shown that resveratrol has biological effects inhibiting breast cancer cell proliferation and tumor invasion and migration [8, 9]. Lucas et al. showed that resveratrol and piceatannol upregulated PD-L1 expression in breast and colorectal cancer cells via HDAC3/p300-mediated NF-*κ*B signaling [10]. Ho et al. indicated that integrin alphavbeta3 mediated the effects of dihydrotestosterone and resveratrol on breast cancer cell proliferation [11]. However, in spite of the biological activities of natural resveratrol such as antitumor, anti-oxidation, and inducing cell apoptosis, its application is constrained by the poor druggability, fast metabolism, target selectivity, and low bioavailability. Studies have shown that structural modification of natural active lead compounds to improve their pharmacological effects is a new strategy for drug development. Previously, a cancer therapeutic conjugate involving Res and tamoxifen citrate by layer-by-layer (LbL) nanoparticles was produced based on lipid-based drug delivery systems (LbDDS) and liquid crystalline nanoparticles (LCNPs), which was a promising means in anti-cancer therapy even if it was at an early application phase [12]. Mesoporous silica material is a new inorganic biomaterial with ultra-high specific surface area, large pore volume, morphology, and size controllable [13]. Mesoporous silica nanomaterials (MSN) have the dual characteristics of mesoporous materials and nanomaterials with very high chemical stability, biocompatibility, convenient synthesis, and low cost, so its applied research in the field of biomedicine has attracted widespread attention [14, 15]. So far, MSN has become an excellent drug delivery system and drug sustained release and Res [16]. As Summerlin et al. indicated, colloidal MSN enhanced resveratrol's biological activity [17]. Recently, MSN-Res is indicated to suppress gastric cancer progression with better efficacy compared with Res treatment alone, which reveals the potential of MSN-Res to improve drug delivery in antitumor therapy [18]. However, the function of Res coated by MSN on breast cancer remains unclear.

These results suggested that MSN loaded by Res has better performance on breast cancer therapy. Thus, we intend to enhance the antitumor effects of Re by synthesizing MSN-Res and the treatment efficacy in breast cancer was further explored.

## 2. Materials and Methods

### 2.1. MSN-Res Preparation

MSN-Res preparation was based on the previous report [19]. Briefly, a round-bottom flask was added with 20 mL surfactant solution (2.0/20) and placed in an oil bath constantly stirred and heated to 95 °C. Then triethanolamine (TEA; 100 *μ*L) was supplemented and stirred the solution for 1 h, followed with the dropwise addition of tetraethylorthosilicate (TEOS; 1.5 mL) and maintained for 6 h. MSNs were collected by centrifugation at 13,000 rpm for ten minutes and rinsing by ethanol. Next, 1% sodium chloride solution was used to extract the solution for 4 h. Finally, the calcination of MSNs was performed using a muffle furnace for ten h at 550 °C to remove the complete surfactant. Furthermore, rotary evaporation technique was used to load Res [20]. The commercialized MSNs and Res were purchased from Nanjing Nonoeast Biotechnology (Nanjing, China) and Yuanye Biotechnology Co., LTD (Shanghai, China).

### 2.2. Characterization of Res-Loaded MSN

Pore size distribution was calculated by the ASAP 2000 analysis program using the method by Barrett, Joyner and Halenda (BJH) [21]. The surface area was determined by a nitrogen adsorption analyzer (BELSORP-mini, Japan). X-ray diffraction (XRD, STOE, Germany) was used to physiochemically examine the nanoparticles. Nanoparticles were suspended in deionized water at 2, 4, 6, 8, and 10 pH values, and Zetasizer Nano Plus instruments were used for measurements of zeta potential. Cytotoxicity assay was performed by cell proliferation *in vitro* and H&E staining *in vivo*.

### 2.3. Cell Culture and Treatment

The human breast cancer cell line MGF-7 was purchased from ATCC and was incubated in the 90% Dulbecco's modified eagle medium (DMEM; Thermo Fisher, Shanghai, China) containing 10% FBS (Sigmal-Aldrich, Shanghai, China) and 100 U/mL penicillin and 100 *μ*g/mL streptomycin with 5% CO_2_ at 37 °C in a humidified incubator. MGF-7 cells at 70-90% confluence were treated with or MSN-Res with different concentrations.

### 2.4. Cell Counting Kit-8 (CCK-8) Assay

CCK-8 was used to detect cell proliferation according to the manufacturer's instructions. Briefly, MGF-7 cells with different treatments were harvested, washed, and resuspended with 100 *μ*L fresh complete medium at 0, 24 h, 48 h, and 72 h after incubation. Next, 10 *μ*L of CCK-8 solution (Dojindo, Kyushu, Japan) was supplemented to culture with the cells at 37 °C and 5% CO_2_ for 1 h. The optical density (OD) value was measured by the microplate reader at 450 nm.

### 2.5. Cell Migration Measurement

Wound healing assays were used to evaluate cell migration ability. When cell confluence reached 90%, a 200-*μ*l pipette tip was used to scratch the cell monolayer to create a wound. The wound was observed and imaged at 0 h and 24 h by a microscope, and the wound width was quantified using ImageJ software.

### 2.6. Transwell Invasion Assay

The MGF-7 cells with different treatments were harvested and resuspended in a serum-free DMEM. Next, 1 × 10^5^ cells were seeded into the upper transwell chamber precoated with Matrigel, and the lower chamber was inserted into a well containing 20% serum from a 24-well plate. The cells on the upper membrane surface were cleaned using a cotton swab after incubation for 24 h. The cells adhering to the lower membrane surface were fixed with 4% paraformaldehyde and stained with 0.1% crystal violet. Furthermore, invading cells were counted under an optical microscope.

### 2.7. Flow Cytometry

MGC-7 cells were plated in six-well plates and treated with Res and MSN-Res, respectively, at the confluence reached 70%-90%. The cultured cells were harvested by trypsinization after 48-h incubation. Cell apoptosis was detected using FITC Annexin-V and PI Apoptosis Detection Kit (BD, USA). Briefly, the cells were washed twice with cold phosphate-buffered saline (PBS) and then resuspended with binding buffer to 1.0 × 10^5^ cells/100 *μ*L. And then, the cells were stained with 5 *μ*l of FITC Annexin-V and 5 *μ*l of propidium iodide (PI), and the apoptosis cells were analyzed by flow cytometry.

### 2.8. Animal Experiments

The BALB/c nude mice (male, 3-4 weeks, 20-25 g) were provided by Shanghai Model Organisms (Shanghai, China) and bred under 60%-65% humidity at 22-25 °C in 12/12 h light/dark cycles. For tumor-bearing mice of MGC-7 cells establishment, a total of 3 × 10^6^ cells was injected into the flanks of nude mice subcutaneously. Next, the animals were randomly separated into 3 groups into the Control, Res, and MSN/Res groups with 5 mice in each group. The tumor size of the tumor was monitored and measured on day 28 after injection. All the experiments were approved by the Ethics Committee and the ethics of Xinhua Hospital Chongming Branch, School of Medicine, Shanghai Jiaotong University.

### 2.9. Hematoxylin and Eosin (H&E) Staining, Immunohistochemistry, and Terminal-Deoxynucleotidyl Transferase Mediated Nick End Labeling (TUNEL)

After fixing the tumor tissue samples in 10% formalin, and cut into 7-*μ*m slices, the sections were stained with hematoxylin and eosin (G1005, Servicebio, China) for H&E staining. For Ki-67 immunohistochemistry, Anti-Ki-67 antibodies were added to the sections and cultured overnight at 4 °C, followed with incubation with the matched secondary antibody for 2 h. Subsequently, DAB substrate was used to stain the sections. And then, the sections were dehydrated and sealed with coverslips after counterstaining with hematoxylin. For the TUNEL assay, the section staining was performed using a TUNEL kit. After being dewaxed and hydrated, sections were treated with proteinase K solution for 15 min, followed with the treatment of DNaseI reaction solution (100 *μ*L). After incubation with 100 *μ*L of TdT enzyme reaction solution for 1 h at 37 °C in the dark and then incubated with 100 *μ*L of streptavidin-HRP for 30 min at 37 °C, a high-capacity digital slide scanner system was applied to analyze the sections.

### 2.10. RNA Extraction and Reverse-Transcription Quantitative PCR (RT-qPCR)

Based on the manufacturer's protocol, total RNA isolation was performed using Trizol reagent (Invitrogen, CA, USA). Complementary DNA (cDNA) was reversed using a ReverTra Ace qPCR RT Master Mix with a gDNA Remove kit (Toyobo, Japan). According to the manufacturer's instructions, RT-qPCR was conducted using an SYBR Premix Ex Taq II (Takara, Japan) on an ABI7500 real-time PCR System. Glyceraldehyde-3-phosphate dehydrogenase (GAPDH) is used as an internal control. We used 2^-△△Ct^ methods to calculate the relative expression of genes. The primers used in the present study were as follows:

F-IkB-5′-CCTGACCTGGTTTCGCTCTT-3′, R-IkB-5′-AGGTAAGCTGGTAGGGGGAG-3′;

F-p65-5′-CGCTGCGGAGCTTGTAGTC-3′, R- p65-5′-GTTCCTGGTCCTGTGTAGCC-3′;

F-GAPDH-5′-GGGTCCCAGCTTAGGTTCATC-3′, R-GAPDH-5′-TACGGCCAAATCCGTTCACA-3′.

### 2.11. Western Blotting

Protein was extracted from tissues by radioimmunoprecipitation assay (RIPA) lysis buffer. And then, the quantity of the proteins was quantified using a BCA Protein Assay Kit. 20 *μ*g protein was loaded on 10% SDS-polyacrylamide gel electrophoresis (SDS-PAGE) gel and then transferred onto polyvinylidene difluoride (PVDF) membranes (Millipore). Then the membranes underwent coincubation with the primary antibodies IkB, P65, p-IkB, p-P65, and GAPDH (Abcam, Shanghai, China) at 4 °C overnight after blocking with 5% nonfat milk for 1 h at 37 °C. After rinsing thrice by TBST buffer, membranes were incubated with secondary horseradish peroxidase-goat anti-rabbit/mouse antibodies (Abcam). The protein bands were detected using enhanced chemiluminescence (ECL) following the manufacturer's instructions.

### 2.12. Statistical Analysis

The data in the present study were analyzed by the SPSS version 22.0 statistical software, and the results were exhibited as the mean ± standard deviation. Student's *t*-test or two-way analysis of variance (ANOVA) were used to analyze the difference among the two or more two groups. *P* < 0.05 indicated a significant difference.

## 3. Results

### 3.1. Characteristics of MSN-Res

**Figure 1:**
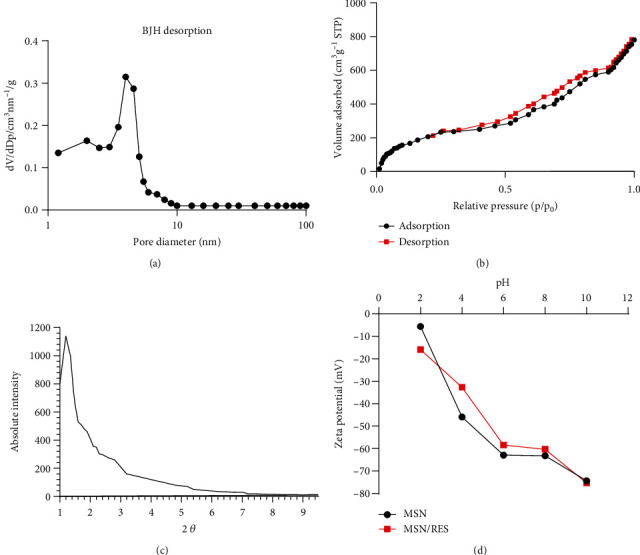
Characteristics of MSN-Res. (a), The distribution of pore sizes of MSN-Res. (b), The isotherms of nitrogen adsorption/desorption in MSN-NH2. (c), MSN-Res XRD patterns revealed the peak location related with the mesoporous silica structure. (d), MSN-Res surface charge was analyzed by zeta potential measurements.

The BET nitrogen adsorption/desorption method was used to determine the surface area, pore pattern, and pore volume of MSN-Res. The pore diameter for MSN-Res is 5 nm as revealed by the BJH method (Figure 1(a)). The BET surface area of MSN-Res was 437 m^2^/g (Figure 1(b)). The XRD pattern of MSN-Res demonstrated the retention of the 2D hexagonal shape of nanoparticles with a p6 mm symmetry (Figure 1(c)). Finally, zeta potential analysis showed the MSN-Res surface charge under gradient pH values. A negative surface charge was identified in pristine and Res-loaded particles (Figure 1(d)), which indicated the minor influence of Res loading on MSN zeta potential values.

### 3.2. Cytotoxicity of MSN-Res

**Figure 2:**
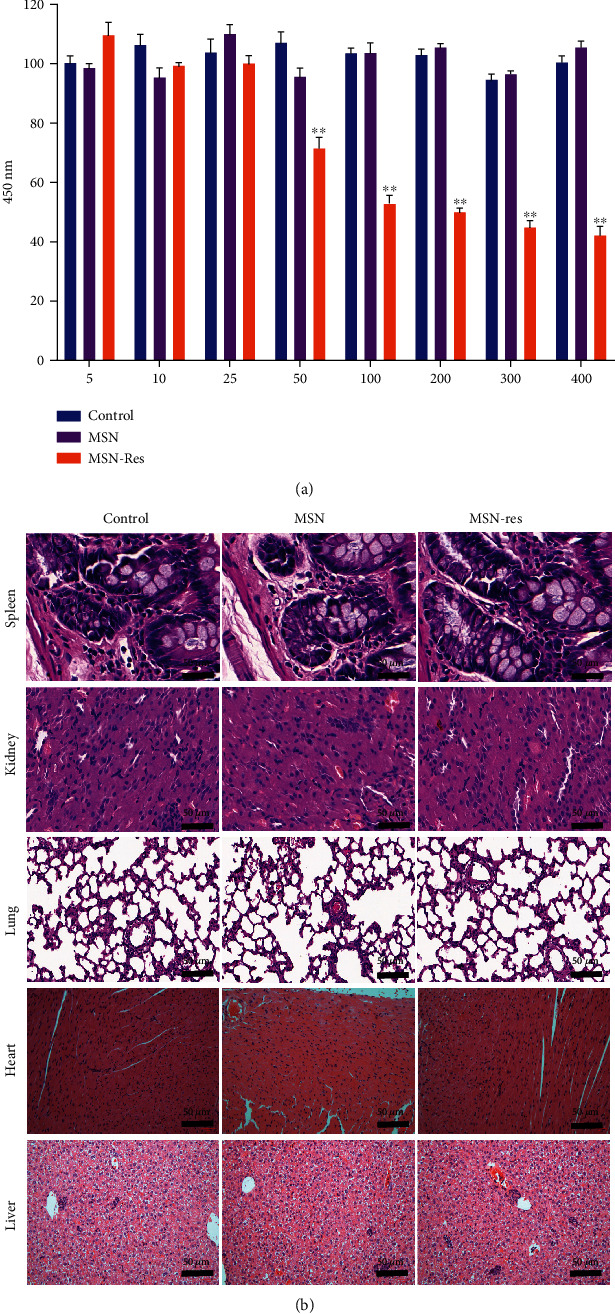
Cytotoxicity of MSN-Res. (a), Cell proliferation potential after treatment with MSN or MSN-Res with different concentrations; (b), H&E analysis was conducted to evaluate the effect of MSN or MSN-Res on mouse organs *in vivo* (scale bar: 50 *μ*m).

The cytotoxicity of MSN-Res was detected in MGF-7 cells and xenograft mouse models. As shown in Figure 2(a), cell proliferation was not affected as the concentration of MSN or MSN-Res was less than 25 *μ*g/mL compared with the control group. Histological analysis for the lung, heart, liver, spleen, and kidney *in vivo* showed no apparent inflammatory cell infiltration and was observed in the MSN-Res or Res groups (Figure 2(b)). These results indicated that MSN-Res demonstrates sufficient biosafety *in vitro* and *in vivo*.

### 3.3. MSN-Res Inhibits MGF-7 Cell Proliferation, Invasion, and Migration and Promotes Apoptosis

**Figure 3:**
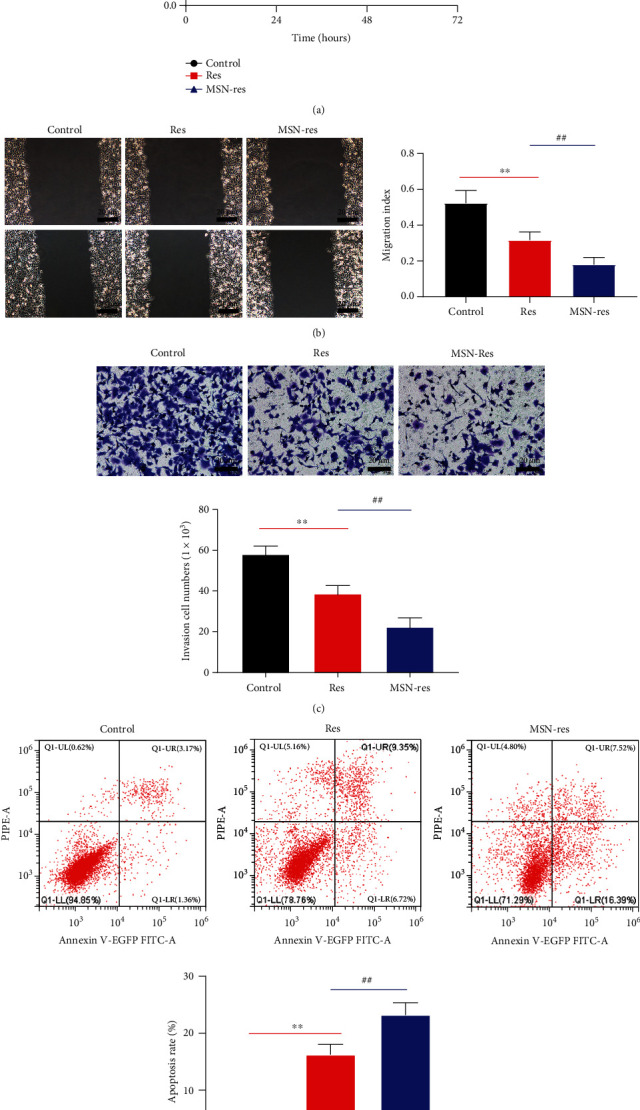
MSN-Res inhibited MGF-7 cell proliferation, invasion, and migration and promoted apoptosis. (a), Cell proliferation ability after treatment with Res or MSN-Res was assessed by cell counting kit-8 assays. (b), The migration of MGF-7 cells affected by Res or MSN-Res was subject to wound healing assays. (c), Transwell assay was used to detect the invasion of MGF-7 cells administration with Res or MSN-Res (scale bar: 20 *μ*m). (d), Flow cytometry was used to detect the apoptosis rate after treatment with Res or MSN-Res. ^∗∗^*P* < 0.01 vs. Control; ^##^*P* < 0.01, MSN-Res group vs. Res group.

Functional experiments were conducted to evaluate the values of MSN-Res against breast cancer. The results demonstrated that cell proliferation potential was dramatically suppressed in the Res or MSN-Res groups in comparison with the control (Figure 3(a)). Wound healing and transwell assay showed that the invasion and the migration were dramatically inhibited post administration with Res or MSN-Res (Figures 3(b) and 3(c)). Flow cytometry showed that the apoptosis was significantly promoted in the Res or MSN-Res treatment groups (Figure 3(d)). Furthermore, MSN-Res exerted better inhibitory effects on the malignant cell behaviors of MGF-7 cells than in the Res group (Figures 3(a)-3(d)). Overall, MSN-Res inhibits MGF-7 cell proliferation, invasion, and migration and promoted apoptosis.

### 3.4. MSN-Res Inhibits Breast Cancer Progression with Better Performance than Res Alone

**Figure 4:**
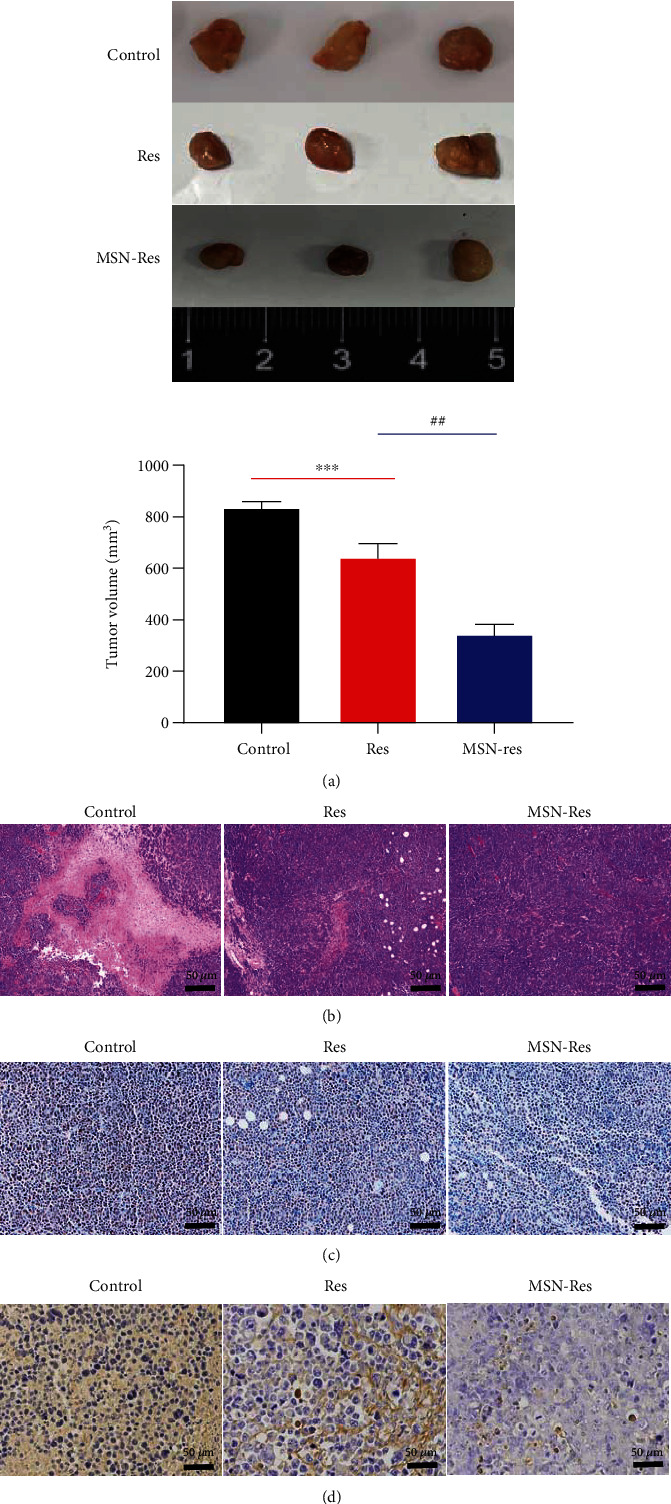
The effect of MSN-Res on breast cancer progression *in vivo*. (a) The tumor size affected by MSN-Res or Res. (b) H&E analysis was conducted to assess the inflammatory injury affected by MSN-Res or Res (scale bar: 50 *μ*m). (c) Ki-67 immunohistochemical staining was applied to measure MGF-7 cell proliferation when treatment with MSN-Res or Res (scale bar: 50 *μ*m). (d) TUNEL analysis was applied for cell apoptosis evaluation after indicated treatments (scale bar: 50 *μ*m). ^∗∗^*P* < 0.01 vs. Control; ^##^*P* <0.01, MSN-Res group vs. Res group.

In addition, we detected the function of MSN-Res in breast cancer murine models after injection with MGF-7 cells. As shown in Figure 4(a), both Res and MSN-Res could reduce the tumor size in comparison with the control group. Furthermore, the tumor size was further reduced in treatment with MSN-Res compared with the Res group. H&E analysis indicated that MSN-Res and Res could alleviate inflammatory injury and the degree of attenuation is significantly higher in the MSN-Res group than in the Res group (Figure 4(b)). Furthermore, we found that the proliferation of MGF-7 cells was reduced in the MSN-Res or Res groups than the control, and the inhibitory effect was enhanced by MSN-Res in comparison with the Res (Figure 4(c)). Moreover, MSN-Res or Res could significantly increase the apoptosis than the control, and it was further increased in the MSN-Res group than the Res group (Figure 4(d)). These results suggested that MSN-Res showed better performance in breast cancer treatment than Res *in vivo*.

### 3.5. MSN-Res Inhibits Tumor Growth via Inhibiting the NF-*κ*B Signaling Pathway

**Figure 5:**
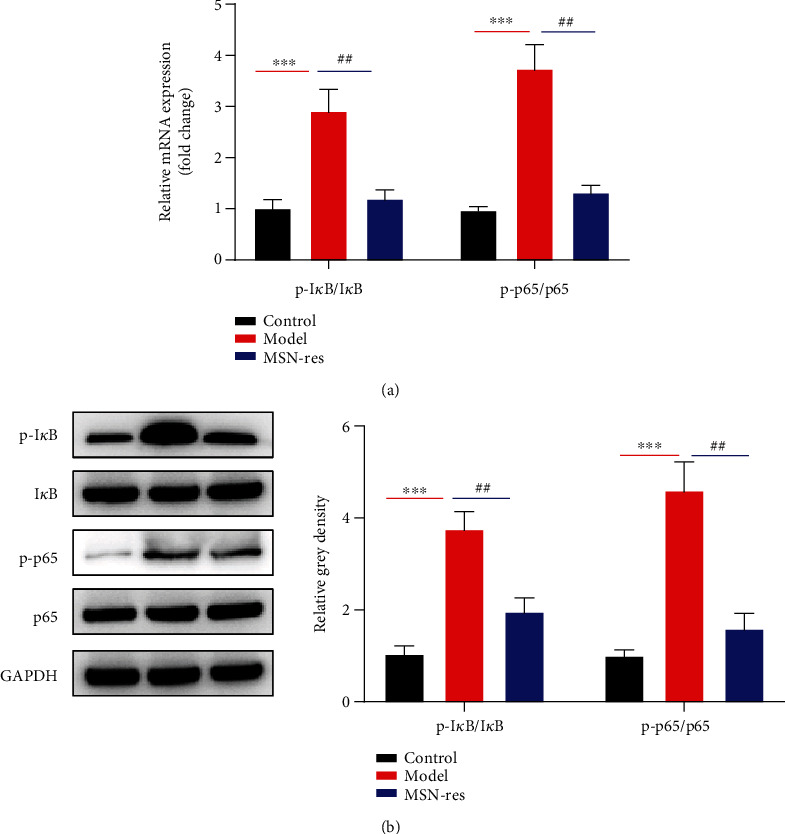
MSN-Res inhibited tumor growth via inhibiting the NF-*κ*B signaling pathway. (a) RT-qPCR was used to detect the expression of IkB and p65 in the model group affected by MSN-Res at mRNA level; (b) western blot was used to detect the protein expression of IkB, p65, p-IkB, and p65, GAPDH acts as the Control. ^∗∗^*P* < 0.01, MSN-Res group vs. Control group.

NF-*κ*B signaling pathways have been involved in various biological processes in breast cancer development. In order to know whether Res affected NF-*κ*B pathway, the IkB and p65 expression was detected. As shown in Figure 5(a), RT-qPCR analysis showed that IkB and p65 were significantly upregulated in the model group compared with the control group. However, the upregulation of IkB and p65 could be decreased when treatment with Res-loaded MSNs. These results were also confirmed by western blot. The results showed that although the total protein of IkB and p65 was not changed, the expression of p-IkB and p65 exhibited significant elevation in the model group. In contrast, MSN-Res could reduce p-IkB and p65 (Figure 5(b)), suggesting that Res inhibited breast cancer tumor progression by inhibiting the NF-*κ*B signaling pathway.

## 4. Discussion

With the in-depth research on the pathogenesis of breast cancer, the comprehensive treatment of breast cancer has been dramatically improved [22]. In recent years, multiple treatment methods, including local surgical treatment of breast cancer, radiotherapy and chemotherapy, endocrine therapy, targeted molecular reagents, and traditional Chinese medicine adjuvant therapy, have significantly improved patients' quality of life and prolonged the survival period [23, 24]. Res is a non-flavonoid polyphenol compound with anti-aging, antitumor, anti-inflammatory, and immune-regulating effects [25, 26]. The anticancer effect of Res has been revealed in the previous studies. For example, Res inhibits migration and metastasis of breast cancer by reversing TGF-beta1-induced epithelial-mesenchymal transition [27]. Yang et al. indicated that Res enhances the cisplatin induced inhibition on cell migration and invasion and tumor growth both *in vitro* and *in vivo* in breast cancer MDA-MB-231 cell models [27]. Özdemi et al. showed that Res increased the sensitivity to cisplatin for the MDA-MB-231 cell line by regulating intrinsic apoptosis [28]. However, Res has the characteristics of insoluble in water, poor stability, low bioavailability, short half-life, fast metabolism, and elimination rate, etc.; its clinical application is greatly restricted. Therefore, it is vital to overcome these limitations to better its application in breast cancer treatment.

According to the first report on the anticancer effects of TAM/Res-loaded LCNPs on human breast cancer cells, TAM/Res–LbL-LCNPs were therapeutically efficient, safe, and biocompatible for human red blood cells with no noticeable toxicity, adverse side effects, or behavioral abnormalities in mice implying their safety in biomedical applications [12]. Thus, it is feasible and effective to seek a new and safer way of using phytochemicals in combinatorial therapy. Mesoporous silica (MSN) is a nanocarrier with a high specific surface area, adjustable particle size and pore size, and good biocompatibility. These characteristics make it have excellent application prospects in catalysis, nanoreactor, drug slow-release, chemical sensing, etc. For example, Summerlin found that colloidal MSN could enhance Res's biological activity [17]. Estelle et al. indicated that Res encapsulation with MSN did not alter its bioactivity. Res encapsulation provided higher anti-inflammatory activity than both resveratrol suspension and solution, suggesting the potential of small size MSN as nanocarriers for hydrophobic drugs and nutraceuticals [29]. It has been widely used in the study of drug delivery systems and cancer therapy and Res. A study has revealed that Anti-miR21 and MSNs loaded with Res conjugated with hyaluronic acid (HA) are developed to improve the efficacy of gastric cancer treatment [30]. Recently, Diogo et al. indicated that Res-loaded MSN showed good encapsulation and enhanced release of Res from MSN for melanoma therapy [19]. We successfully prepared the Res-loaded MSN in the present study, which showed high biosafety. Further analysis indicated that MSN loaded with Res significantly inhibits breast cancer cell growth *in vitro* and tumorigenesis *in vivo*, suggesting the values of MSN to improve the Res delivery for breast cancer treatment.

Nuclear transcription factor-kB (NF-*κ*B) is a multidirectional nuclear transcription factor that can regulate the expression of a variety of cytokines and enzymes. NF-*κ*B is critically implicated in various kinds of biological processes in breast cancers, including cell migration, invasion, adhesion, and epithelial-mesenchymal transition [31–33]. Meanwhile, several studies showed that Res could affect NF-*κ*B signaling pathways in different diseases. As Yi et al. showed, Res alleviated the interleukin-1*β*-induced chondrocytes injury through the NF-*κ*B signaling pathway [34]. Shang et al. indicated that Res exhibited protective effect on the myocardium against sepsis via the activation of the PI3K/AKT/mTOR signaling and inhibition of the NF-*κ*B pathway [35]. Pozo-Guisado et al. showed that Res-caused apoptosis of MCF-7 cells is related to a caspase-independent mechanism by downregulating NF-*κ*B and Bcl-2 expression [36]. Lucas et al. showed that Res and piceatannol upregulated PD-L1 expression in breast and colorectal cancer cells through the NF-*κ*B signaling [10]. Inconsistent with these findings, we revealed that MSNs loaded with Res could reduce p-IkB and p65, suggesting that Res inhibited breast cancer tumor progression by inhibiting the NF-*κ*B signaling pathway.

## 5. Conclusions

MSN-Res inhibited breast cancer progression with improved efficacy than Res treatment alone by inhibiting the NF-*κ*B signaling pathway, suggesting that MSN-Res is a more effective adjuvant treatment method for breast cancer, which provides a new and safer means of using phytochemicals in combinatorial therapy of breast cancer.

## Data Availability

Original data included in this study were available from the corresponding authors upon reasonable requests.
